# Use of copper-functionalized cotton waste in combined chemical and biological processes for production of valuable chemical compounds

**DOI:** 10.1039/d3ra06071c

**Published:** 2023-11-28

**Authors:** Michal J. Binczarski, Justyna Z. Zuberek, Payam Samadi, Malgorzata Cieslak, Irena Kaminska, Joanna Berlowska, Aleksandra Pawlaczyk, Malgorzata I. Szynkowska-Jozwik, Izabela A. Witonska

**Affiliations:** a Lodz University of Technology, Institute of General and Ecological Chemistry 116 Zeromskiego Street 90-924 Lodz Poland michal.binczarski@p.lodz.pl; b Lukasiewicz Research Network – Lodz Institute of Technology, Department of Chemical Textile Technologies 19/27 Marii Sklodowska-Curie Street 90-570 Lodz Poland; c Lodz University of Technology, Department of Environmental Biotechnology 171/173 Wolczanska Street 90-924 Lodz Poland

## Abstract

Cotton textiles modified with copper compounds have a documented mechanism of antimicrobial action against bacteria, fungi, and viruses. During the COVID-19 pandemic, there was pronounced interest in finding new solutions for textile engineering, using modifiers and bioactive methods of functionalization, including introducing copper nanoparticles and complexes into textile products (*e.g.* masks, special clothing, surface coverings, or tents). However, copper can be toxic, depending on its form and concentration. Functionalized waste may present a risk to the environment if not managed correctly. Here, we present a model for managing copper-modified cotton textile waste. The process includes pressure and temperature-assisted hydrolysis and use of the hydrolysates as a source of sugars for cultivating yeast and lactic acid bacteria biomass as valuable chemical compounds.

## Abstract 2

Scheme of processing with Cu functionalized cotton waste.

## Graphical abstract

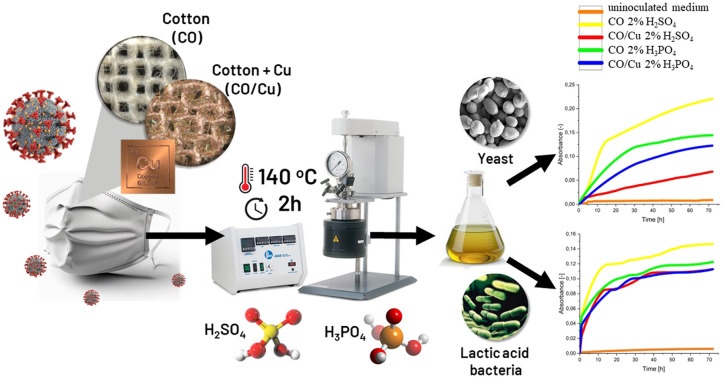

## 1. Introduction

Textile materials made of natural, chemical, and synthetic fibers can be functionalized to produce bioactivity against bacteria, viruses, and fungi. Functionalization with bioactive agents can improve the resistance of textiles against biodeterioration and biofouling. Functionalized textiles are widely used in many medical, hygiene, cosmetic, and technical products.^1–6^ The market for functionalized textile products is growing rapidly. In particular, new modifiers and biofunctionalization methods are sought to reduce the risk of SARS CoV-2 and Healthcare-Associated Infections (HAIs).^7^ Most research is focused on the development of textile materials with antibacterial and antiviral properties.^8–16^ One interesting method is the use of metallic particles, including copper and its compounds and complexes.^17–19^

Copper compounds are among the bioactive modifiers most widely used to functionalize textiles.^20,21^ Copper-based modifiers have a well-documented mechanism of antimicrobial activity against bacteria, fungi, and viruses.^22–29^ Copper is also used to impart conductive properties, for example in Textronics or photocatalytic materials.^30–33^ A Scopus search for “copper & textiles” returned 2485 publications for the years 1924–2022 (first quarter). Until 2010, the annual numbers of publications on these topics were in the low tens. During the COVID-19 pandemic, they increased sharply to 229 in 2020 and 246 in 2021. There was particular interest in the biomodification of textile structures for use as bio-barrier materials (*e.g.* masks, special clothing, surface coverings, tents, *etc.*).^34,35^

All textile products eventually become waste. Therefore, there is widespread interest in developing technologies to completely transform textile waste into useful economic products, including chemicals and energy fuels.^36–38^ According to the principles of sustainable development and the circular economy, functionalized antimicrobial cotton waste should be managed to reduce pollution. Currently, most textile waste is landfilled,^39,40^ burned,^41–43^ reprocessed, or recycled.^44^ In 2020, both China and America recycled 15% of their textile materials, while the United Kingdom recycled 27%, and Denmark recycled the most textile material at 40%.^45^ It is difficult to determine the scale of the amount of copper textile waste in the literature, but the global wearable technology market is forecast to grow from $116.2 Bn in 2021 to $265.4 Bn by 2026. Electronic textiles (e-textiles) have recently attracted increasing interest, as they complement the mainstream sector of general wearable electronics. This type of material has a wide range of applications. It can be used in clothing and protective gear, as well as for medical, decorative, and technical purposes. Electronic textiles can also be used in agro-textiles and functional accessories.^12,46–49^ There are currently no studies dedicated to copper functionalised cotton waste treatment methods. This is probably related to the general problem of separating special textile streams from waste. However, it is likely that developments in the functionalisation of textiles, including cotton, will make it necessary to improve the selectivity of waste collection.^50^

Our previous research showed that cotton waste subjected to catalytic hydrolysis can be repurposed as a good medium for microorganisms, including yeasts and bacteria. Yeasts and bacteria can metabolize the simple sugars present in hydrolysates to produce energy products, such as ethanol and methane.^51,52^ Although cotton waste can be reused or recycled in a rational and environmentally friendly way, the toxicity of copper presents a significant challenge. Copper-functionalized textile waste can have a detrimental effect on the environment if not managed correctly.^53–57^ Because they are relatively new on the textile market, there has been little research on processing copper-functionalized materials. The catalytic conversion of cotton modified with Cu into valuable fermentation media may constitute a new direction for dealing with this type of waste. The biotechnological products, including lactic acid and ethanol, can be further processed into valuable chemicals and intermediates for synthesis. Our research is therefore anticipatory, indicating one possible use of such waste.

Numerous studies have shown that the toxicity of copper depends on its concentration and form. In a study by Sun *et al.*,^58^ copper ions in concentrations of up to 32 mg L^−1^ did not inhibit the growth of the yeast strain *Saccharomyces cerevisiae* BH8. Only higher concentrations inhibited yeast growth and resulted in significant cell death. Similar results were reported by Azenha *et al.*^59^ for the *Saccharomyces cerevisiae* DL1 strain. However, when Avery *et al.*^60^ investigated the toxicity of copper against the *Saccharomyces cerevisiae* NCYC 1383 strain, they found that a significant decrease in yeast viability occurred only at a concentration of about 300 mg L^−1^, as a result of the loss of cell membrane integrity. According to the literature on the toxicity of copper ions to lactic acid bacteria strains, copper should not inhibit lactic acid fermentation.^61^ However, the presence of Cu^2+^ ions in the hydrolysis reaction medium may result in the formation of other oxidation/reduction products of sugars, apart from glucose, which could be potential inhibitors of fermentation processes.

This article presents research showing the potential repurposing of copper-modified cotton waste as an additive in fermentation media for yeast and lactic acid bacteria. These microorganisms produce ethanol and lactic acid and can also be used as high-protein feed additives after thermal degradation of cells. The article demonstrates the potential reuse of copper-modified cotton waste as a fermentation media additive for yeast and lactic acid bacteria. These microorganisms produce ethanol and lactic acid, and can be used as high-protein feed additives after thermal degradation of the cells. The demand for lactic acid is increasing year on year. In 2022, about 1.5 million tons of lactic acid were produced, and this amount is expected to double in the next 10 years.^62^ Lactic acid plays a significant role in major industries, including the food industry (as a food preservative, fermenting agent, acidifying agent, antioxidant, *etc.*), the chemical industry (as a decalcifying agent, pH regulator, green solvent, cleaning agent, *etc.*), the cosmetics industry (as a moisturizing agent, brightening agent, rejuvenating agent, *etc.*), and the pharmaceutical industry (as a dialysis solution, mineral preparation, tablets, *etc.*).^63–65^ The proposed solution for the treatment of textile waste has a double benefit: the method allows the generated waste to be processed, while the glucose can be used for the production of valuable chemical compounds in biotechnological processes. Preliminary research was conducted into the possibility of using thermally assisted acidic hydrolysis to treat copper-modified cotton waste. We investigated the susceptibility of the cotton waste to release glucose in the process of thermal acid hydrolysis with H_2_SO_4_ and H_3_PO_4_ acids. The physicochemical characteristics of the copper-functionalized cotton waste were analysed using optical microscopy, scanning electron microscopy coupled with X-ray micro-area analysis (SEM-EDS), and inductively coupled plasma coupled with mass spectrometry (ICP-MS). The quantitative and qualitative composition of the hydrolysates was determined, paying attention to the formation of potential fermentation inhibitors (HPLC, GC-MS). We then evaluated the ability of yeast and lactic acid bacteria to multiply on media containing the hydrolysates.

## 2. Experiment

### 2.1 Materials and methods

#### 2.1.1 Characteristics of cotton fabric and copper functionalized cotton fabric

Table 1 lists the characteristics of the raw cotton woven fabric (CO) used in the study. The raw cotton fabric was modified on one side using a DC magnetron sputtering system (P.P.H. Jolex s. c., Czestochowa, Poland)^66^ with copper target purity of 99.99%. Sputtering with Cu was carried out using the following parameters: argon content 3%; effective power 2.0–2.2 kW h; circulating power 0.8–1.0 kW; speed 15 mm s^−1^; 15 passages. Functional CO/Cu fabric was obtained.^12^

**Table 1:** Characteristics of raw cotton woven fabric (CO)

		Cotton fabric (CO)
Raw material		Cotton 100%
Fiber transverse size, μm		15.52 ± 1.81
Yarn	Warp	Tex 8
	Weft	Tex 8
Wave		Plain
Treads number/10 cm	Warp	310
	Weft	300
Mass per unit area, g m^−2^		54.75

**Fig. 1:**
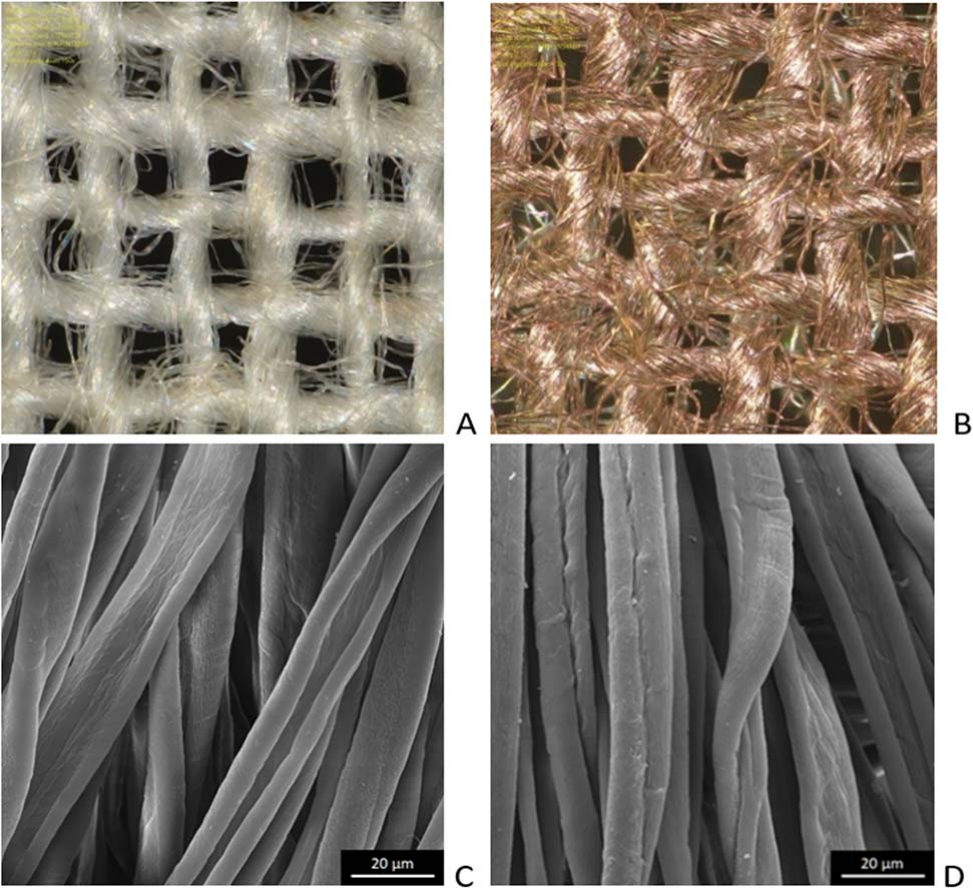
Microscopic images of cotton fabrics: CO (A) and CO/Cu (B), magnification ×150. Scanning electron microscope (SEM) images of cotton fibers: CO (C) and CO/Cu (D), magnification ×2000.

Microscopic images of the raw CO and CO/Cu fabrics were taken using an OLYMPUS DSX1000 digital optical microscope (OLYMPUS, Tokyo, Japan) (Fig. 1A and B) and a VEGA3 scanning electron microscope (SEM) (TESCAN, Brno, Czech Republic).

SEM/EDS analysis of CO and CO/Cu fabrics was performed using an Energy Dispersive Spectrometer (EDS) INCA X-ray microanalyzer (Oxford Instruments Analytical, High Wycombe, United Kingdom) coupled with a scanning electron microscope VEGA3. X-ray microanalysis was conducted under air pressure of 10 Pa, with an accelerating voltage of 20 kV and a BSE backscattered electron beam, using the SmartMap function. For Cu modified fabric, total spectrum maps of the distribution of carbon (C), oxygen (O), and copper (Cu) were prepared, including weight percentages. Maps were determined for the Kα line with excitation energy *E* = 0.28 keV (C), *E* = 0.52 keV (O), and *E* = 8.04 keV (Cu).

#### 2.1.2 Characteristics of chemicals and reagents

The reagents and chemicals used in the study are summarised in Table 2.

**Table 2:** Summary of reagents and reagents

Name	Concentration	Company	Country	Purity class
Copper target	99.99%	Testbourne Ltd.	UK	Analytical gr.
Sulfuric(vi) acid	95%	Stanlab	PL	Analytical gr.
Phosphoric(v) acid	85%	Stanlab	PL	Analytical gr.
Ammonia solution	25%	POCh	PL	Analytical gr.
Acetonitrile	—	Chempur	PL	HPLC gr.
Nitric acid	65%	Baker Analyzed	PL	Analytical gr.
Indium solution	—	Merck	USA	ICP class
MEB medium	—	Merck	USA	Technical gr.
YPG medium	—	BTL sp. Z o. o.	PL	Technical gr.

#### 2.1.3 Characteristics of the hydrolysates of cotton fabric and copper-functionalized cotton fabrics (CO and CO/Cu fabrics)

Samples containing 1 g of ground CO or CO/Cu fabrics were subjected to thermally assisted acid hydrolysis in a pressure reactor with a volume of 50 mL (Parr Instrument Company 4552 Series Mini Reactor, Moline, Illinois, USA). Hydrolysis reactions were carried out at 140 °C for 2 h with 2% sulfuric(vi) acid – or 2% phosphoric(v) acid (85%, Stanlab, Lublin, Poland). The reaction mixtures were cooled in the reactor to room temperature, neutralized by the addition of NH_4_OH to pH 7–7.5, and filtered to remove solid yarn residues using a funnel with a sintered disc lined with a quality hard filter (Filtrak, Ahlstrom-Munksjö Group, Helsinki, Finland). The designations of the fabrics and the conditions of hydrolysis are given in Table 3.

**Table 3:** Designations of the hydrolysates obtained after acid hydrolysis of CO and CO/Cu fabrics

Designations of hydrolysates	Fabrics	Acid	Temperature [°C]	Time [h]
1	CO	2% H_2_SO_4_	140	2
2	CO/Cu		140	2
3	CO	2% H_3_PO_4_	140	2
4	CO/Cu		140	2

The process conditions (*T*, *C*_H_3_PO_4_, H_2_SO_4__, *t*) were optimized in a previous study by Binczarski *et al.*^52^

The glucose concentrations in the hydrolysates were determined using high-performance liquid chromatography (HPLC, Sykam GmbH, Eresing Germany, with an S1125 pump system, S 5300 autosampler, S 4115 column thermostat and RI S 3585 detector). The sugars were separated on a SETREX IEX-H^+^ column (300 × 8.0 mm ID) at 80 °C using 0.008 mol per L H_2_SO_4_ + 2% v/v ACN (flow 0.8 mL min^−1^) as the mobile phase. Quantitative analysis of glucose was performed on the basis of a calibration curve plotted for the concentration range of 0–10 g L^−1^ (the curve in the analyzed range was linear *y* = 0.19733*x*, *R*^2^ = 0.9998803).^52^

The non-sugar compounds in the hydrolysates were measured by GC-MS (Shimadzu GC-2010 with an MS-QP2010SE detector and a ZB-5MSplus capillary column, dimensions 30 m × 025 mm × 0.25 μm, with helium as a carrier gas (Linde; 99.999%)). The operating conditions for GC-MS analysis were as follows: flow rate of the carrier gas 8.8 mL min^−1^; ion sources temperature 200 °C; interference temperature 250 °C; column temperature 50–150 °C; initial temperature 50 °C; start time 3 min; temperature rise 5 °C min^−1^; hold time 5 min; injection volume 1 μL.

#### 2.1.4 Elemental analysis of CO and CO/Cu fabrics and their hydrolysates

All the studied material were weighed in glass tubes on an analytical balance with accuracy of four decimal places. The average mass of the solid samples was around 0.15 g. The average mass of the liquid samples was approximately 3 g. All the samples were treated with 4 mL of concentrated nitric acid (65% HNO_3_, Baker Analyzed). The contents of the tubes were mixed with concentrated nitric acid, closed with a Teflon tip, and placed in a Milestone UltraWAVE microwave-assisted digestion system. A blank sample was prepared in the same way. An inert gas (nitrogen) was pumped into the stainless steel reactor under pressure of 40 bar. The chosen sample decomposition procedure consisted of two stages:

Stage I: duration 10 min. The temperature in the reactor was gradually increased up to 230 °C. Maximum pressure inside the reactor 120 bar. Maximum power 1500 W.

Stage II: duration 15 min. The temperature was maintained at 220 °C during the whole stage. Maximum pressure inside the reactor 150 bar. Maximum power 1500 W.

Samples after decomposition in the microwave oven were quantitatively transferred to clean 25 mL volumetric flasks. Indium solution was added to all samples to a final concentration of about 0.4 mg L^−1^, as an internal standard to monitor reproducibility in a given matrix. In all samples of cotton fabrics and their hydrolysates enriched in a standard indium solution, indium concentrations were measured using the ICP-OES technique. The assumed concentrations of this internal standard were obtained, which confirmed the correctness of the applied measurement method. The ICP-OES instrument (iCAP 7400, Thermo Scientific) was calibrated using a multielement standard solution of Merck IV with an initial concentration of 1000 mg L^−1^ of Cu and In.

The highest standard concentration of the analyzed element was 10 mg L^−1^. The lowest was 0.002 mg L^−1^. The samples with the highest levels of Cu were diluted an additional three times, then measured. The position of the torch was horizontal (axial) for lower levels of Cu or vertical (radial) for higher levels of Cu. The choice of emission lines was made based on their relative intensity and the types of interference. The concentration values were finally calculated against the following emission lines: Cu 324.754 nm (axial or radial); In 325.609 nm (axial). The results were verified by analysis of the WatR Supply Metals 697 (Eraqc) certified reference material for river water. There was satisfactory agreement between the results obtained for Cu and the certified value (1.0 mg L^−1^). All the data were within the QC performance acceptance limits (the average recovery values were above 95%). The reproducibility of the internal standard (In) in the matrix of the analyzed samples was also satisfactory.

#### 2.1.5 ICP-OES iCAP 7400, Thermo Scientific technical parameters

Sample introduction system: 4-channel peristaltic pump, concentric glass nebulizer, glass cyclonic spray chamber; plasma gas 12 L min^−1^; auxiliary gas 0.5 L min^−1^, nebulizer gas 0.5 L min^−1^; plasma viewing: axial or radial; RF source 27.12 MHz. Spectrometer: simultaneous echelle type 52.91 grooves per mm ruled grating, 383 mm, effective focal length, 9.5° UV fused silica cross dispersion prism; spectral bandpass 7 pm at 200 nm; wavelength range 166–847 nm; detector: high performance solid-state CID chip; internal standard: In; number of replicates: 3.

#### 2.1.6 Morphology of solid CO and CO/Cu residues after acid hydrolysis

A scanning electron microscope (SEM S-4700, Hitachi, Tokyo, Japan) equipped with an energy dispersive spectrometer (EDS, Thermo-Noran Inc., Madison, WI, USA) was used for SEM analysis of the hydrolysates. The samples were embedded in conductive carbon pads. The excess loose powder was removed. To reduce electric charging, the samples were sputter coated with gold (Cressington 208 HR system). Images were acquired in back-scattered electron (BSE) mode. An accelerating voltage of 25 kV was used. For the purposes of comparison, the same samples were analyzed using an FEI Quanta 650 SEM (FEI Company, Hillsboro, OR, USA) equipped with a Bruker Energy Dispersive Spectroscopy (EDS) system (Bruker Corporation, Billerica, MA, USA). A 15 kV accelerating voltage was used with a 3.5 μA electron beam current and 10 mm working distance. The compositions of each sample were measured at least three times at different locations approximately 0.25 mm^2^ in size.^51^

#### 2.1.7 Cultivation of yeast and lactic acid bacteria on CO and CO/Cu hydrolysates

The obtained CO hydrolysates were used as a component in fermentation medium for the cultivation of yeast and lactic acid bacteria. The hydrolysates were sterilized at 121 °C for 15 min. Then, 180 μL of sterilized medium was transferred into each of the wells on a 96-channel plate and inoculated with 20 μL of an inoculum suspension of yeast cells in MEB medium or an inoculum suspension of lactic bacterial cells in YPG medium. The yeast strains used were *Saccharomyces cerevisiae* Ethanol Red (Fermentis Division S.I., Lesaffre, Marcq-en-Baroeul, France) and *Saccharomyces cerevisiae* Tokay ŁOCK 0204 (ITFiM PŁ collection, Lodz, Poland). The lactic bacteria species used were *Lactobacillus plantarum* AXG (ITFiM PŁ collection, Lodz, Poland) and environmental isolate W3A (ITFiM PŁ collection, Lodz, Poland). Cultivation was carried out at 25 °C (yeast) or 35 °C (lactic bacterial) for 72 h in a Thermo Scientific Multiskan GO UV/VIS spectrophotometer (Thermo Fisher Scientific, Waltham, MA USA) to measure the absorbance of the solutions at a wavelength of 620 nm. The increase in absorbance was proportional to the turbidity of the solution caused by proliferation of the microorganisms.

## 3. Results and discussion

**Fig. 2:**
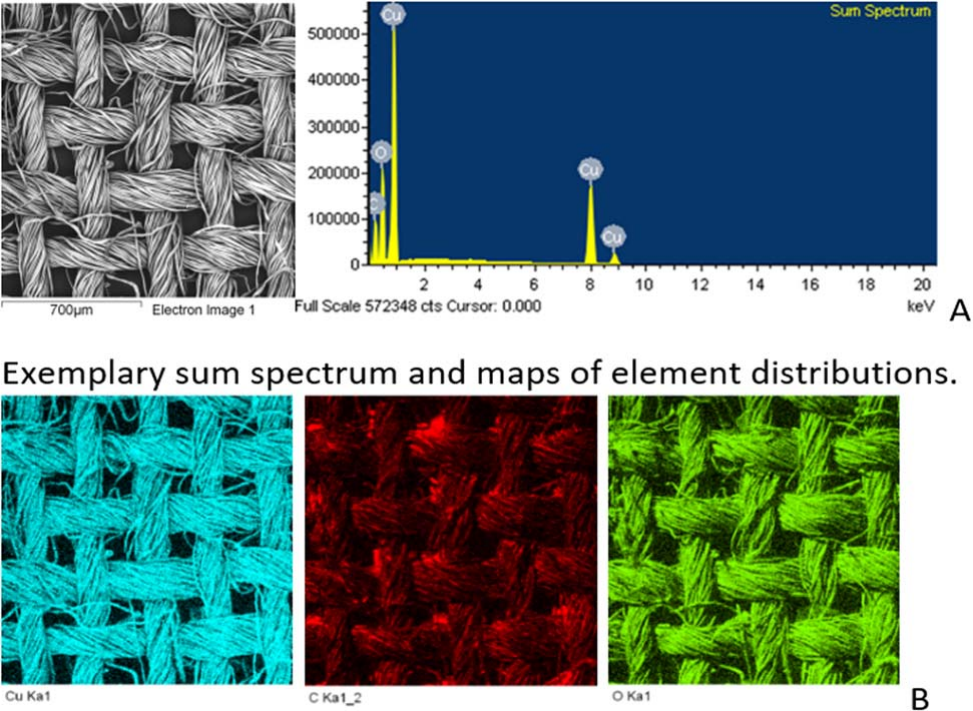
SEM/EDS analysis (A) and weight percentages of elements in CO and CO/Cu fabrics (B).

All cotton (CO) and copper-modified cotton (CO/Cu) fabrics before hydrolysis were characterized using an optical microscope (Fig. 1A and B) and a scanning electron microscope (SEM) (Fig. 1C, D and 2). Additionally, maps of the total distribution spectrum of carbon (C), oxygen (O) and copper (Cu) were prepared for the Cu-modified fabric, taking into account weight percentages (Table 4).

**Table 4:** Mean values of weight percentages of elements, wt%

	C	O	Cu
CO/Cu	28.5 ± 1.67	29.1 ± 0.82	42.4 ± 1.04
CO	47.7 ± 0.25	52.3 ± 0.25	—

After the acid hydrolysis of CO and CO/Cu fabrics, samples of the hydrolysates were neutralized, and the solid residue was separated by filtration. The glucose yield in the liquid hydrolysates was measured using liquid chromatography (HPLC). The results are presented in Table 5.

**Table 5:** Mass of the solid residue after hydrolysis [g] and glucose yield in the hydrolysates [%] (reaction conditions: *T* = 140 °C, *t* = 2 h, cacid = 2%)

Designation of samples	Mass of sample [g]	Mass of cotton in the 1 g of sample^a^ [g]	Mass of cotton in the sample [g]	Mass of the solid residue [g]	Mass of Cu in solid residue^b^ [g]	Mass of the cotton in the solid residue [g]	Loss of cotton weight [g]	Glucose concentration^c^ [g L^−1^]	Glucose yield from 1 gram of sample [g]	Glucose yield [%]
1	1.062	0.998	1.060	0.952	0.007	0.945	0.115	5.70	0.114	99.24
2	0.989	0.764	0.756	0.811	0.199	0.612	0.144	4.15	0.083	57.80
3	1.077	0.998	1.075	0.840	0.009	0.831	0.244	11.98	0.240	98.26
4	0.994	0.764	0.759	0.779	0.295	0.484	0.275	13.31	0.266	96.65

a Cu% – wt in CO sample from ICP measurement = 0.02% (for 1 g sample of CO *m*_Cu_ = 0.002 g); Cu% – wt in CO/Cu sample from ICP measurement = 23.64% (for 1 g sample of CO/Cu *m*_Cu_ = 0.236 g).

b Calculated on the basted of the data listed in Table 7.

c Concentration of glucose in hydrolysates determined by HPLC measurements.

The amounts of glucose obtained from the hydrolysis of CO and CO/Cu fabrics were similar with each acid used. However, the use of H_3_PO_4_ led to the formation of nearly three times more glucose than the use of H_2_SO_4_. This situation is typical for samples of natural materials in which cellulose is the main building material.^52^ On the other hand, using various acids (H_2_SO_4_, H_3_PO_4_) of different strengths may lead to the formation of additional products of hydrolysis, particularly from the cellulose present in cotton. Also, the presence of copper ions in the reaction solution may result in the formation of various products from the released sugars. Therefore, GC-MS analysis of the liquid hydrolysates was performed to identify the non-sugar hydrolysates. The results are presented in Table 6.

**Table 6:** GC-MS analysis of non-sugar compounds in samples of CO and CO/Cu hydrolysates^a^

Compound	Chemical formula	Description of samples			
		1-H	2-H	3-H	4-H
Furfural	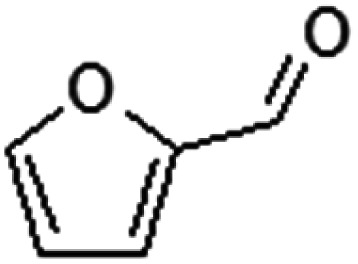	+	+	−	−
Benzoic acid, 2-methoxy-, methyl ester	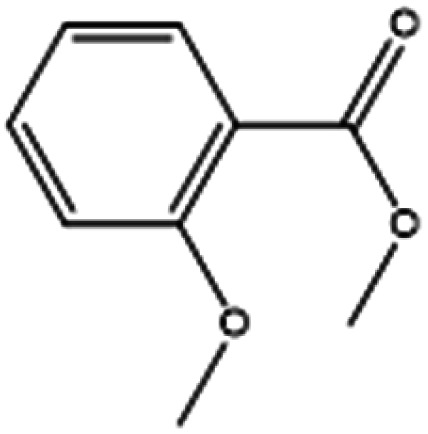	+	+	+	+
2,4-Di-*tert*-butylphenol	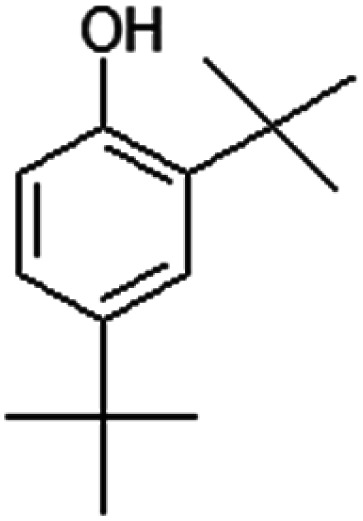	−	+	−	+
Benzoic acid, 4-(dimethoxymethyl)-, methyl ester	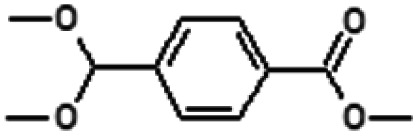	−	−	+	+
Oxime-, methoxy-phenyl	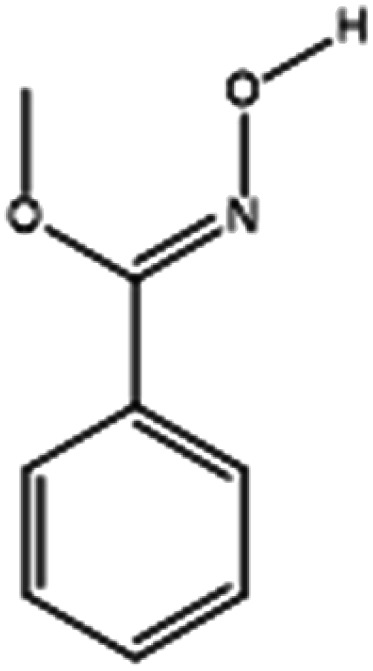	−	−	+	+

a Reaction conditions: *T* = 140 °C, *t* = 2 h, *C*_acid_ = 2%.

When H_2_SO_4_ was used as an acidic catalyst, furfural formed in both the CO and CO/Cu reaction mixtures. Furfural did not form in the reaction mixture when H_3_PO_4_ was used for hydrolysis. However, benzoic acid, 4-(dimethoxymethyl)-, methyl ester, and oxime-, methoxy-phenyl were detected after the reaction. The Cu^2+^ ions released from the cotton fabric during the reaction with acids were probably responsible for the formation of 2,4-di-*tert*-butylphenol during hydrolysis. This compound was present in all the hydrolysates of the copper-modified fabric, regardless of the type of acid used. However, it was not present in the pure cotton hydrolysates. It is worth emphasizing that the amounts of non-sugar products released into the hydrolysates were very low, and probably not sufficient to inhibit the growth of microorganisms.

The copper content in CO and CO/CU hydrolysates and in solid residues after CO and CO/Cu hydrolysis are presented in Table 7. Each sample of copper-modified fabric before hydrolysis contained 23.64% of copper (for 1 g of CO/Cu *m*_Cu_ = 0.236 g). After the hydrolysis process, most of the copper remained in the solid hydrolysis residue, and only a small amount passed into the hydrolysate. This trend was noted for the samples catalyzed with both sulfuric(vi) acid and phosphoric(v) acid. In the hydrolysate sample after hydrolysis catalyzed by phosphoric(v) acid, the copper content was lower than in the case of the sample catalyzed by sulfuric(vi) acid, which proves that H_3_PO_4_ is a weaker acid than H_2_SO_4_, oxidizing metals less. The amounts of Cu^2+^ ions observed in the hydrolysates may inhibit the growth of microorganisms. Sun *et al.*^58^ reported that amounts of copper ions up to 32 mg L^−1^ did not inhibit the growth of *Saccharomyces cerevisiae* BH8 yeast. On the other hand, higher concentrations of copper ions inhibited yeast growth throughout the fermentation process and caused the deaths of large numbers of cells. Similar results were reported for *Saccharomyces cerevisiae* DL1 by Azenha *et al.*^59^ In contrast, Avery *et al.*^60^ reported a marked decrease in the viability of the *Saccharomyces cerevisiae* NCYC 1383 strain with a Cu^2+^ concentration of about 300 mg L^−1^, as a result of the loss of cell membrane integrity. It follows that the hydrolysates obtained in our work may be a good source of sugars for microorganisms, as the concentrations of Cu^2+^ did not exceed the limits for toxicity reported by other authors. The only exception was the hydrolysate obtained using sulfuric acid(vi) as a catalyst, in which the concentration of Cu^2+^ ions was close to 300 mg L^−1^. This hydrolysate may be expected to inhibit the growth of microorganisms when used as a fermentation medium for *Saccharomyces cerevisiae* Ethanol Red and *Saccharomyces cerevisiae* Tokay LOCK0204. The available literature on the toxicity of copper ions in relation to lactic acid bacteria suggests that lactic acid fermentation should not be inhibited by copper.^61^

**Table 7:** Copper content in CO and CO/Cu hydrolysates and in CO and CO/Cu solid residues after hydrolysis [mg L^−1^]^a^

Designation of samples	Mass of sample [g]*	Cu [mg L^−1^]*	% Cu [%]	Mass of Cu in SR after hydrolysis of 1 g of sample [g]	Mass of Cu in H after hydrolysis 1 g of sample [g]
1 – SR	0.148	42.96	0.73	0.007	—
2 – SR	0.157	1548.43	24.52	0.199	—
3 – SR	0.136	57.79	1.07	0.009	—
4 – SR	0.134	4156.53	37.90	0.295	—
1 – H	3.090	3.73	∼0	—	0
2 – H	3.081	297.45	0.23	—	0.046
3 – H	3.041	1.97	∼0	—	0
4 – H	3.059	50.86	0.04	—	0.008

a SR – solid residue, H – hydrolysate, *average results from three measurements, Cu% – wt in CO sample from ICP measurement = 0.02% (for 1 g sample of CO *m*_Cu_ = 0.002 g), Cu% – wt in CO/Cu sample from ICP measurement = 23.64% (for 1 g sample of CO/Cu *m*_Cu_ = 0.236 g).

### 3.1 Scanning electron microscope equipped with energy dispersive spectroscopy SEM-EDS

Table 8 shows SEM pictures of the solid residue after hydrolysis of cotton and copper-modified cotton samples. When H_2_SO_4_ or H_3_PO_4_ were used as catalysts for the hydrolysis of raw CO, the process resulted in degradation of the fiber structure. In the SEM images of the solid residue of the CO fabrics after hydrolysis, shorter fibers with smaller diameters are visible. However, there were no visible differences in terms of appearance between the fibers modified with copper after hydrolysis catalyzed by H_2_SO_4_ or H_3_PO_4_.

**Table 8:** SEM pictures of fibers before and after hydrolysis at 100×, 500×, 1000× magnification

Description of samples	100× magnification	500× magnification	1000× magnification
CO	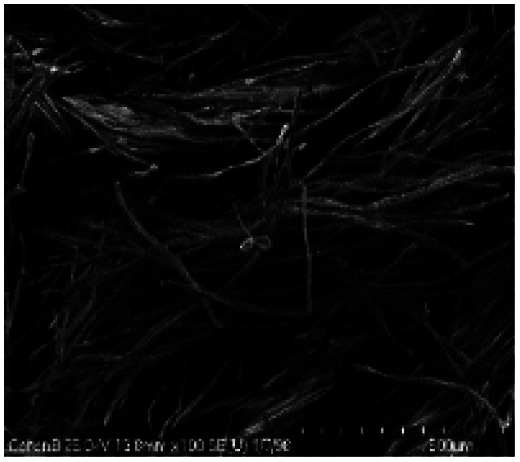	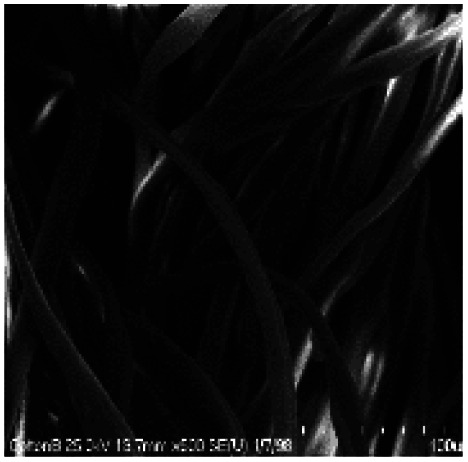	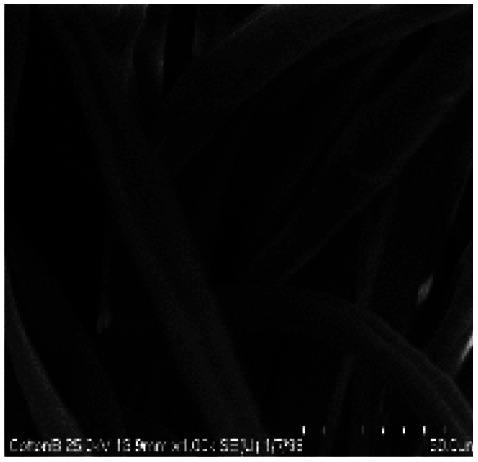
CO/Cu	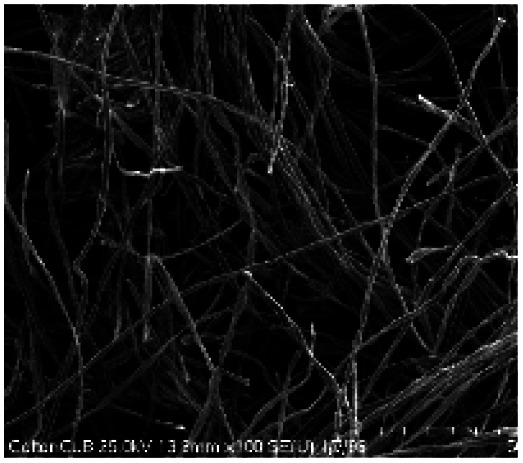	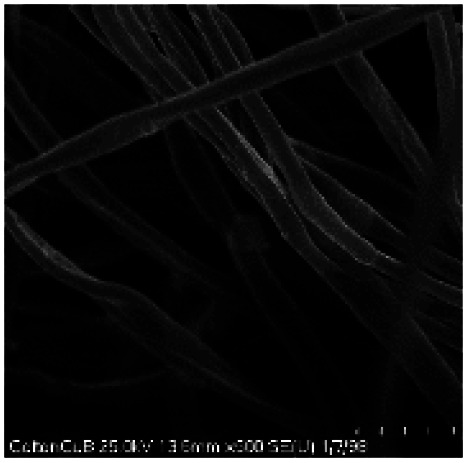	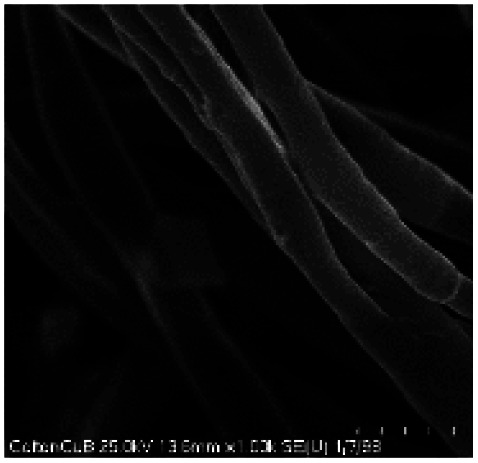
1-SR	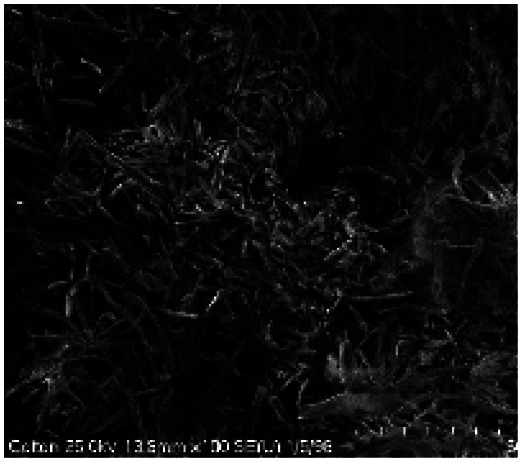	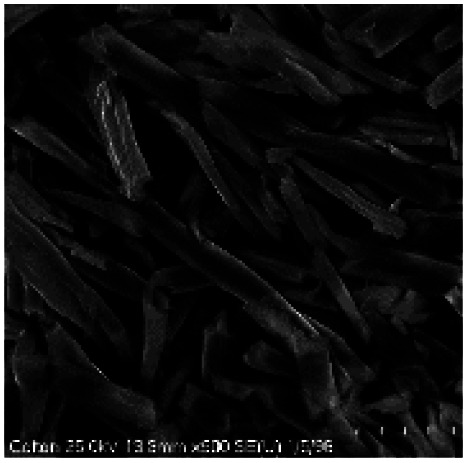	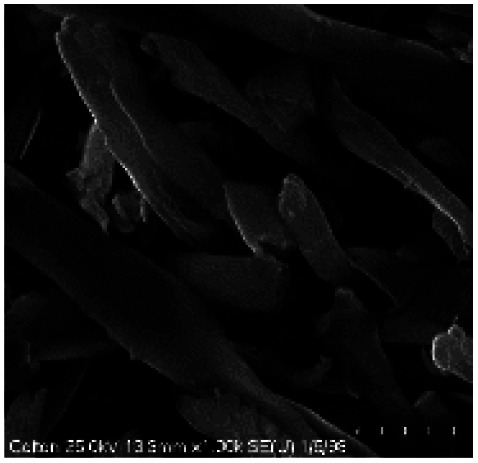
2-SR	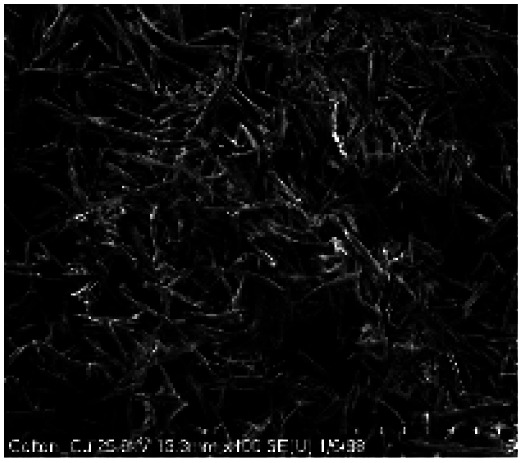	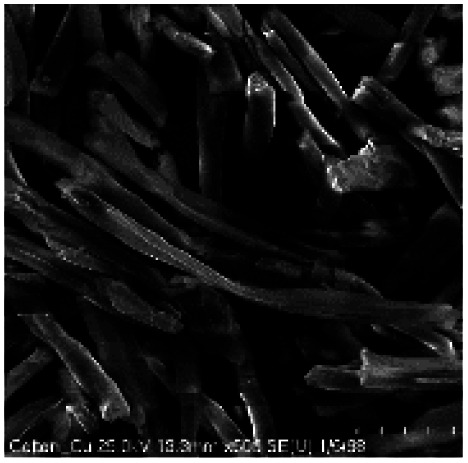	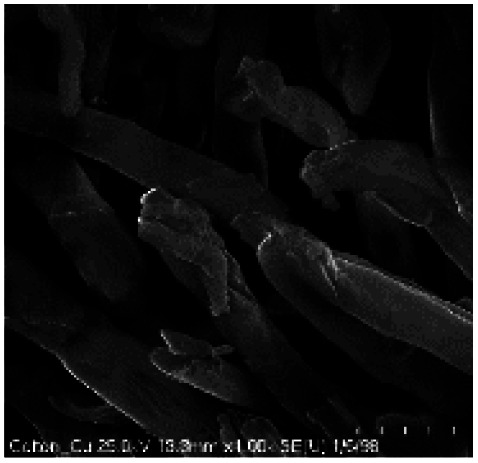
3-SR	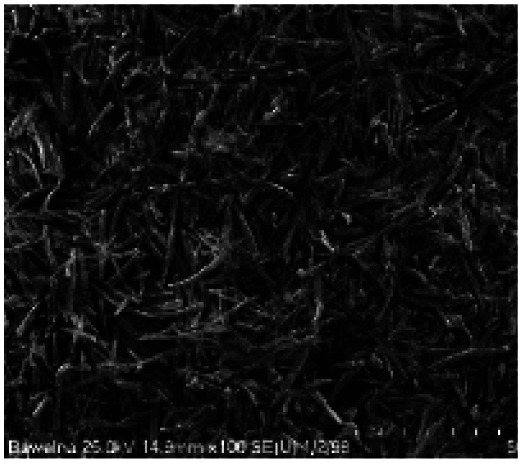	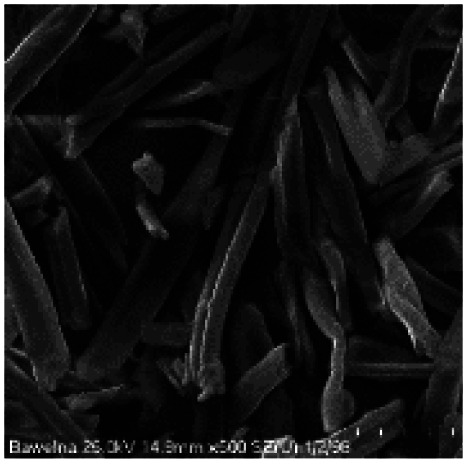	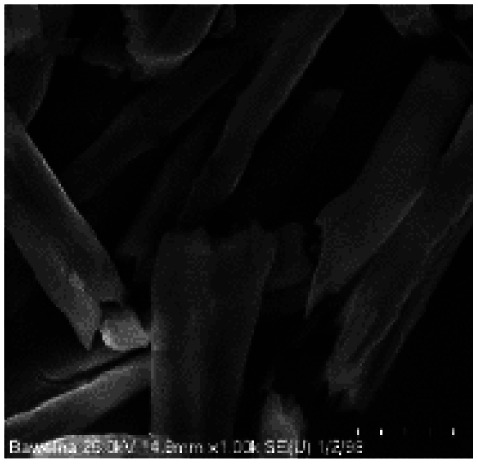
4-SR	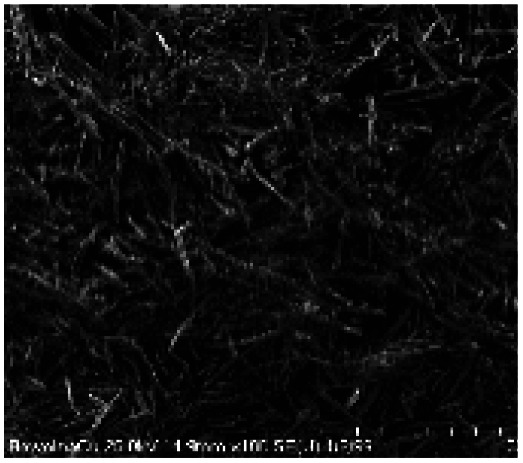	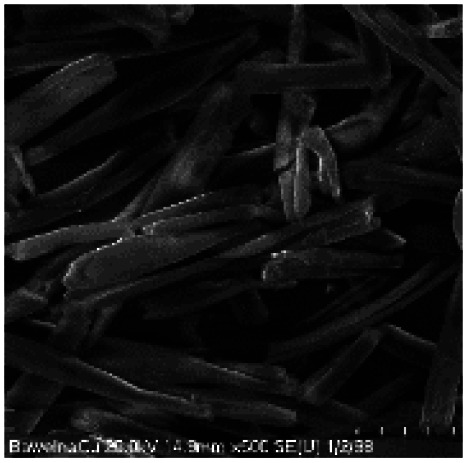	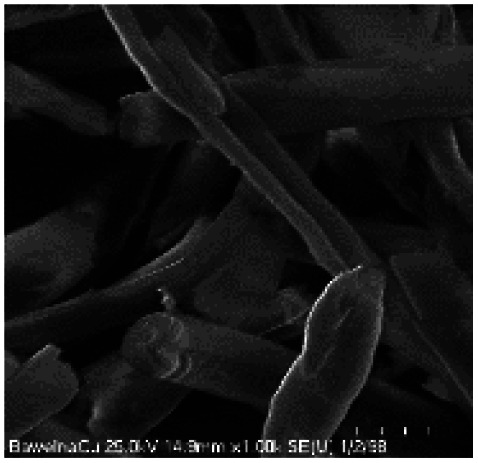

Analysis of the surface compositions of the copper-modified materials after hydrolysis in H_2_SO_4_ or H_3_PO_4_ by EDS confirmed that H_2_SO_4_ had stronger oxidizing properties than H_3_PO_4_, which explains the better solubilization of copper in the hydrolysates catalyzed by H_2_SO_4_. The average weight percentage of copper on the surface of the CO/Cu solid residue was higher for the 2% H_3_PO_4_ catalyzed process than for the 2% H_2_SO_4_ catalyzed process (Table 9).

**Table 9:** Results of EDS analysis (exemplary sum spectra) for CO/Cu solid residue after hydrolysis catalyzed by 2% H_2_SO_4_ and CO/Cu solid residue after hydrolysis catalyzed by 2% H_3_PO_4_, with average values for weight percentage of copper

Sample	Acid used in hydrolysis	Spectra	Average values of Cu wt%
2-SR	2% H_2_SO_4_	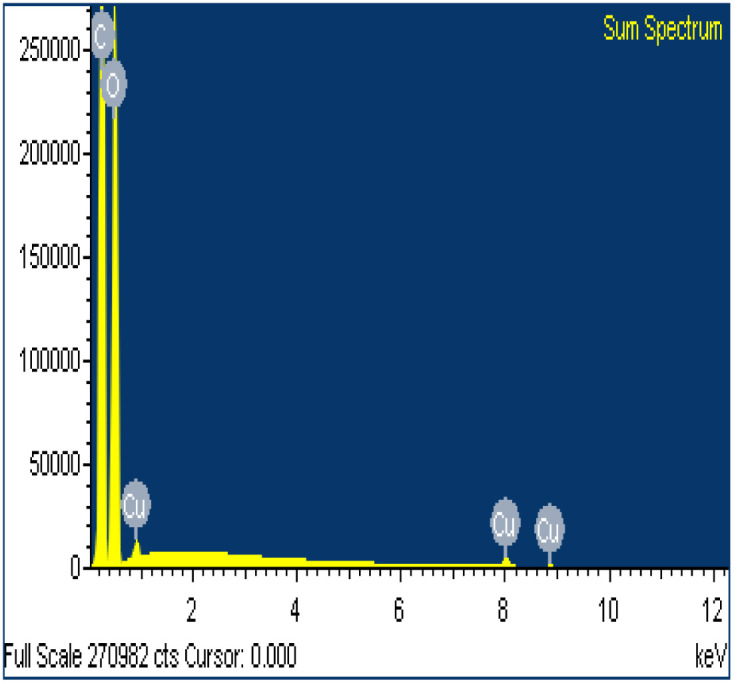	0.71
4-SR	2% H_3_PO_4_	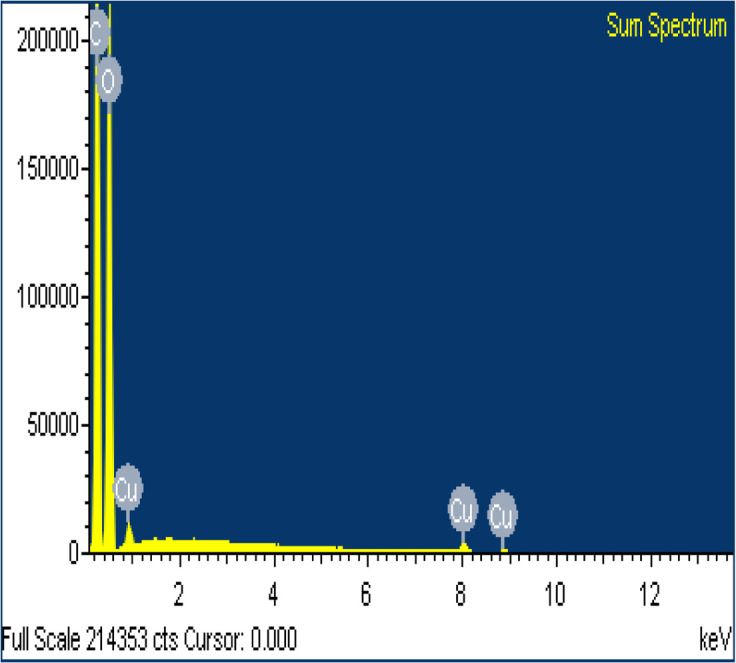	1.18

### 3.2 Yeast and lactic acid bacterial cultivation on cotton hydrolysates

**Fig. 3:**
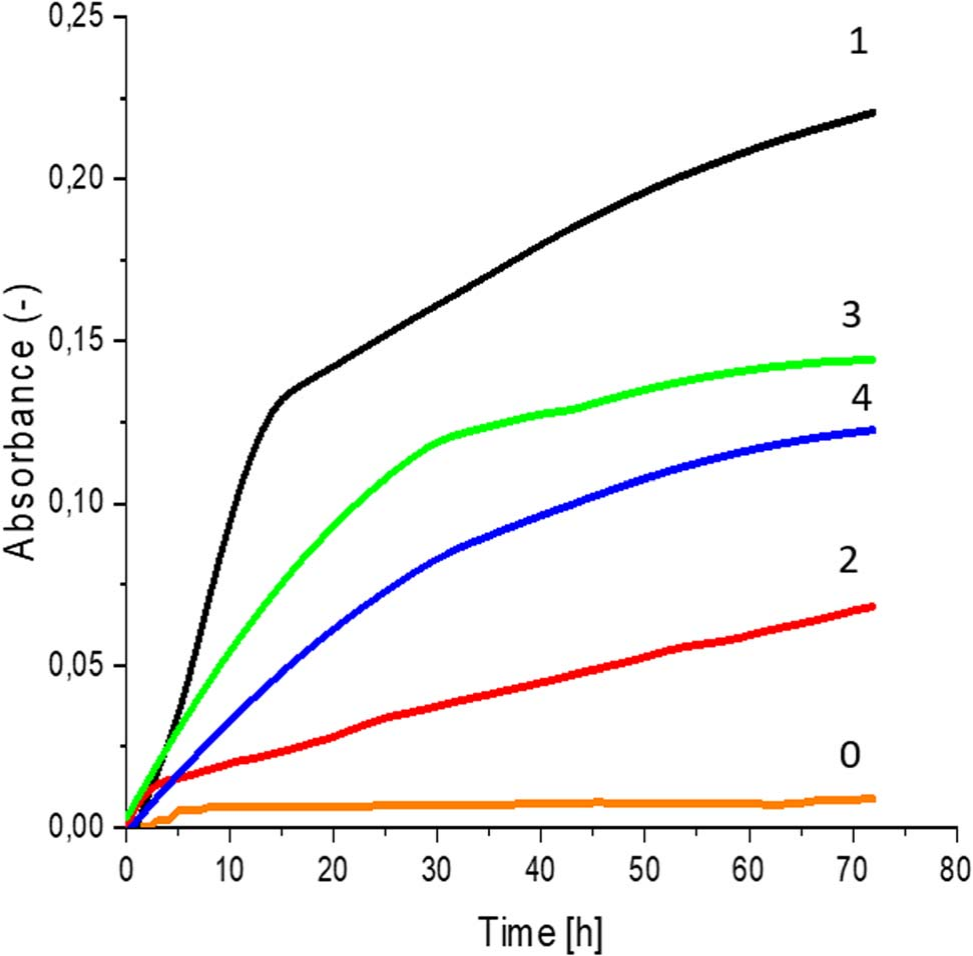
Yeast growth curves for *Saccharomyces cerevisiae* Ethanol Red fermented in: 0 – uninoculated medium (reference sample); 1 – CO hydrolysate, 140 °C, 2 h, 2% H_2_SO_4_; 2 – CO/Cu hydrolysate, 140 °C, 2 h, 2% H_2_SO_4_; 3 – CO hydrolysate, 140 °C, 2 h, 2% H_3_PO_4_; 4 – CO/Cu hydrolysate 140 °C, 2 h, 2% H_3_PO_4_.

**Fig. 4:**
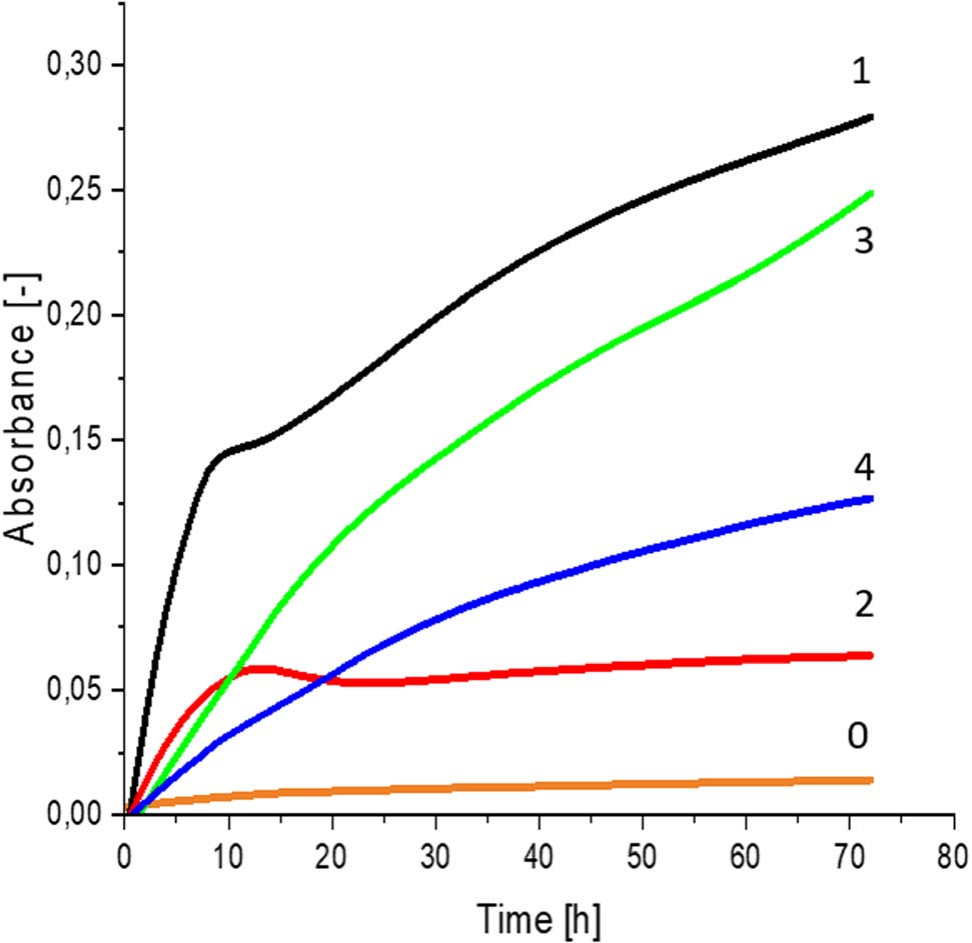
Yeast growth curves yeast growth curves for *Saccharomyces cerevisiae* Tokay LOCK0204 fermented in: 0 – uninoculated medium (reference sample); 1 – CO hydrolysate, 140 °C, 2 h, 2% H_2_SO_4_; 2 – CO/Cu hydrolysate, 140 °C, 2 h, 2% H_2_SO_4_; 3 – CO hydrolysate, 140 °C, 2 h, 2% H_3_PO_4_; 4 – CO/Cu hydrolysate 140 °C, 2 h, 2% H_3_PO_4_.

**Fig. 5:**
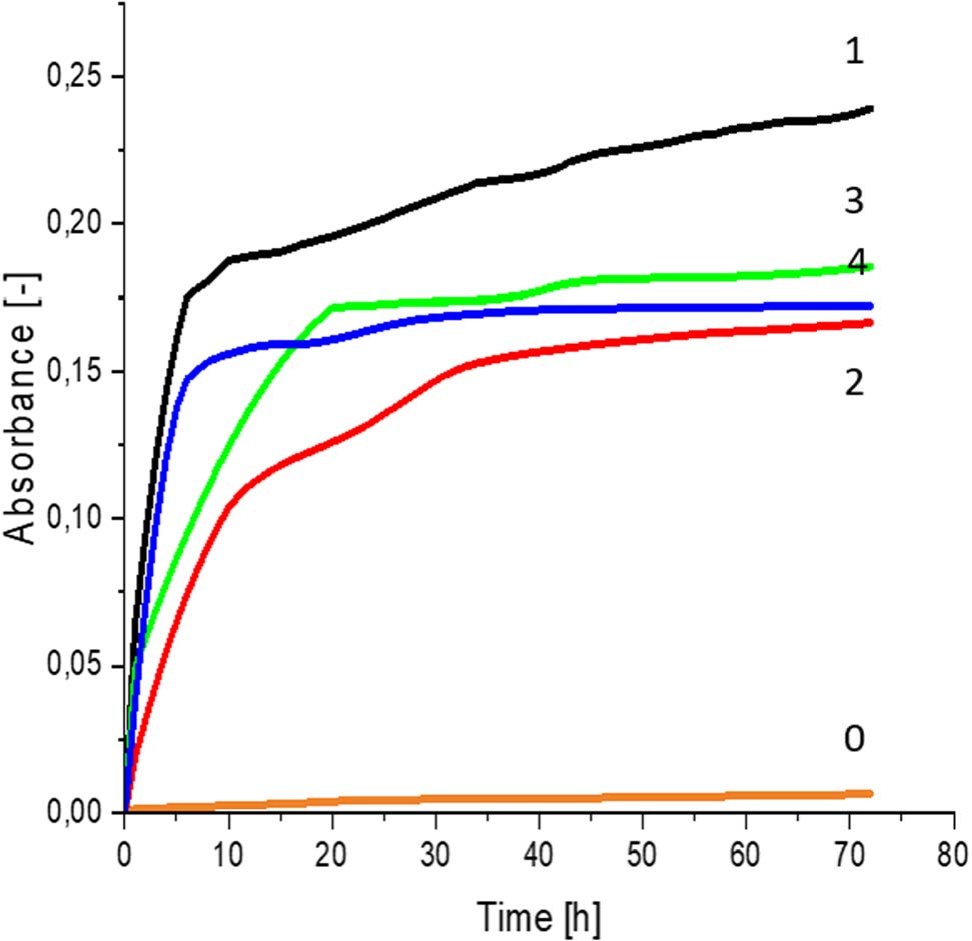
Lactic bacterial growth curves for *Lactobacillus plantarum* AXG fermented in: 0 – uninoculated medium (reference sample); 1 – CO hydrolysate, 140 °C, 2 h, 2% H_2_SO_4_; 2 – CO/Cu hydrolysate, 140 °C, 2 h, 2% H_2_SO_4_; 3 – CO hydrolysate, 140 °C, 2 h, 2% H_3_PO_4_; 4 – CO/Cu hydrolysate 140 °C, 2 h, 2% H_3_PO_4_.

**Fig. 6:**
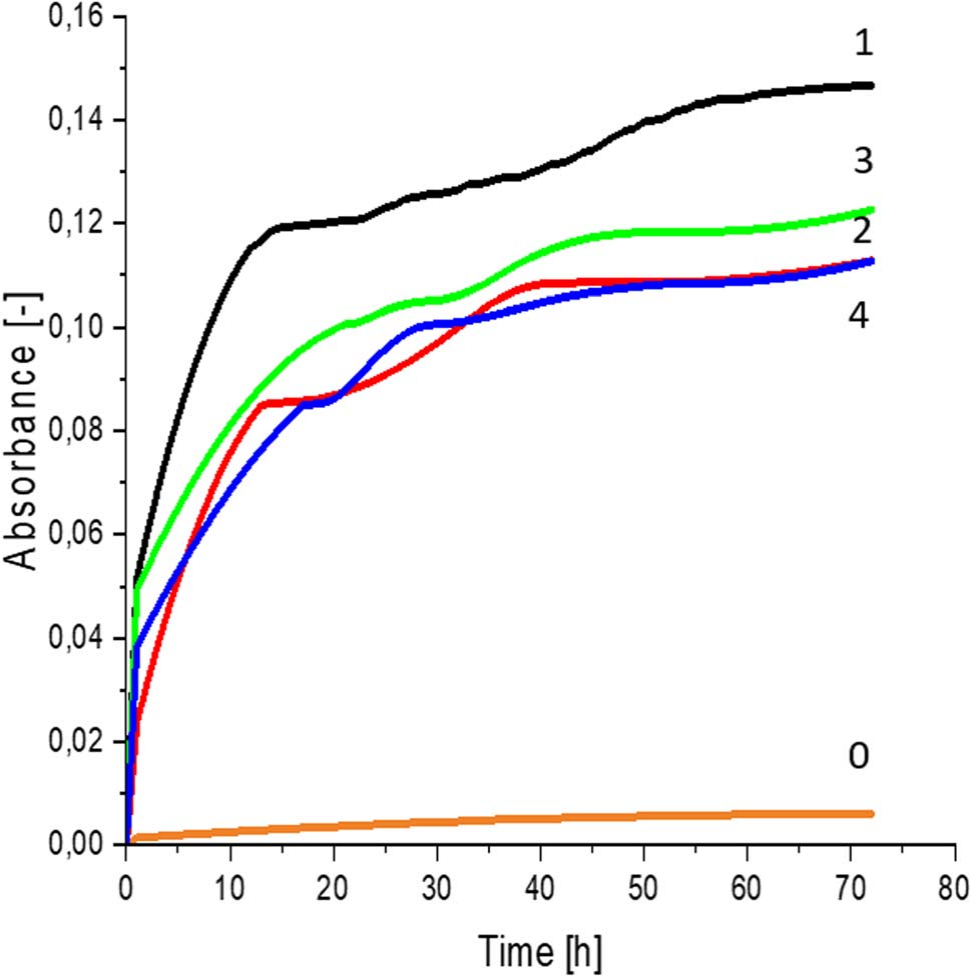
Lactic bacterial growth curves for *Lactobacillus plantarum* W3A fermented in: 0 – uninoculated medium (reference sample); 1 – CO hydrolysate, 140 °C, 2 h, 2% H_2_SO_4_; 2 – CO/Cu hydrolysate, 140 °C, 2 h, 2% H_2_SO_4_; 3 – CO hydrolysate, 140 °C, 2 h, 2% H_3_PO_4_; 4 – CO/Cu hydrolysate 140 °C, 2 h, 2% H_3_PO_4_.

Greater growth of *Saccharomyces cerevisiae* Ethanol Red (Fig. 3) and *Saccharomyces cerevisiae* Tokay LOCK0204 (Fig. 4) yeast cells was noted on CO hydrolysates compared to CO/Cu hydrolysates, in media containing the hydrolysate catalyzed by sulfuric acid(vi) or phosphoric acid(v). When CO and CO/Cu hydrolysates were catalyzed by H_3_PO_4_, the difference in yeast growth was smaller than when they were catalyzed by H_2_SO_4_. This was due to the better decomposition of copper by H_2_SO_4_ than H_3_PO_4_, leading to a higher content of Cu^2+^ ions in the solution, which have an inhibitory effect on yeast growth. Lactic bacteria *Lactobacillus plantarum* AXG (Fig. 5) and *Lactobacillus plantarum* W3A (Fig. 6) on CO or CO/Cu hydrolysates showed little difference in the growth of microorganisms. However, the trend of growth inhibition by Cu^2+^ ions was maintained. This means that lactic acid bacteria are less sensitive to the presence of copper ions in fermentation media. Our results confirm the tendency of copper ions to inhibit the growth of yeast postulated in the literature,^58–60^ as well as the higher tolerance of lactic bacteria to the presence of Cu^2+^ ions.^61^

## 4. Conclusions

Especially since the COVID-19 pandemic, cotton fabrics modified with copper have become commonly used as barrier materials to protect against viruses and bacteria. However, copper-modified cotton fabrics are currently difficult to recycle, as the natural biodegradability of cotton is inhibited. Therefore, new effective methods are needed to neutralize this type of waste and eliminate it from the environment. This study presents the new possibility of combining biological and chemical methods to effectively dispose of copper-modified cotton fabric waste, producing biotechnological products with economic benefits.

**Fig. 7:**
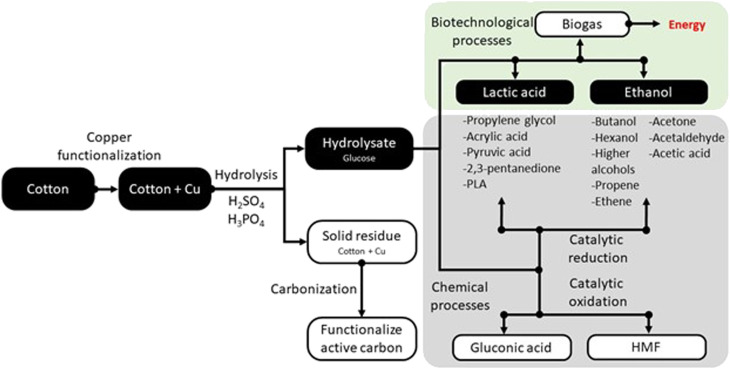
Possibilities for managing copper functionalized textile waste.

Fig. 7 shows the possibilities for managing copper-modified textile waste. The route for obtaining lactic acid and ethanol is marked by black boxes in the diagram. Biotechnological processes enabling the conversion of waste in an environmentally friendly way are highlighted in green and chemical processes are highlighted in gray. The selection of products is justified by the growing demand for lactic acid and ethanol. It is important to use the stream of textile precipitation to obtain these valuable particles. Lactic acid is used extensively in food, feed and cosmetics, as well as in the bioplastics industry. The lactic acid market was valued at approximately USD 2.9 billion in 2021, with an expected annual growth rate of 8%. This platform molecule enables the production of a large pool of valuable compounds, including propylene glycol,^67^ acrylic acid,^68^ PLA,^69^ pyruvic acid,^70^ and 2,3-pentanedione.^71^ Similarly, the wide use of ethanol justifies our decision to process the textile waste in this direction.^72^ Processing the platform molecule makes it possible to obtain butanol,^73^ propene,^74^ ethene,^75^ ethyl,^76^ acetone,^77^ acetaldehyde,^78^ acetic acid,^79^ and large hydrocarbons.^80^ An alternative route may be chemical glucose processing. Examples of such products are HMF^81^ and gluconic acid.^51^ Hydrolysis of copper-functionalized cotton waste leads to two products: glucose hydrolyzate and a solid residue that was not decomposed during the process. The solid residue after appropriate transformation can also be well utilized. An interesting solution for disposing of waste copper-containing material is to transform it into functional activated carbons. Copper modification of the surface of activated carbons increases the selectivity of the adsorption process.^82–84^

## Author contributions

Conceptualization, M. J. B., J. Z. Z., M. C., and I. A. W.; methodology, M. J. B., J. Z. Z., M. C., and I. A. W.; formal analysis, M. J. B., J. Z. Z., M. C., and I. A. W.; investigation, M. J. B., J. Z. Z., P. S., M. C., I. K., J. B., A. P., M. I. S. and I. A. W.; writing—original draft preparation, M. J. B., J. Z. Z., M. C., and I. A. W.; writing—review and editing, M. J. B., J. Z. Z., M. C., and I. A. W.; visualization, M. J. B., J. Z. Z.; supervision, I. A. W.; project administration, I. A. W. All authors have read and agreed to the published version of the manuscript.

## Conflicts of interest

There are no conflicts to declare.

## Supplementary Material
